# Interleukin-31 Receptor α Is Required for Basal-Like Breast Cancer Progression

**DOI:** 10.3389/fonc.2020.00816

**Published:** 2020-05-27

**Authors:** Yanling He, Xinyuan Zhang, Weijun Pan, Fang Tai, Li Liang, Jian Shi

**Affiliations:** ^1^Department of Pathology, Nanfang Hospital & School of Basic Medical Science, Southern Medical University, Guangzhou, China; ^2^School of Basic Medical Science, Guangzhou Medical University, Guangzhou, China

**Keywords:** IL31RA, IL31, Twist, BRD4, BLBC

## Abstract

**Purpose:** Interleukin-31 receptor α (IL31RA) usually mediates IL-31 induced inflammation and allergic diseases. However, the functional roles of IL-31/IL31RA signaling in basal-like breast cancer (BLBC) progression remain totally unclear.

**Methods:** Tumorsphere formation, transwell, and wound healing assays were used to measure the BLBC progression. We implanted tumor cells in mammary fat pad and tail vein of nude mice to detect the growth and metastasis of BLBC cells. Luciferase and ChIP assays were employed to measure the transcriptional regulation. Western blot and real-time PCR assays as well as bio-informatics analyses were conducted to observe the expression of IL31RA.

**Results:** We found that silencing of *IL31RA* suppresses the cancer stem cell-like properties, migration and invasion of BLBC cells *in vitro* as well as tumor growth and metastasis *in vivo*. Knockdown of *IL31RA* ameliorates IL-31-mediated pro-oncogenic functions. Overexpression of IL31RA in luminal breast cancer cells enhances the cancer stem cell-like properties and cell motility. Our data further identified IL31RA as a target gene of Twist/BRD4 transcription complex.

**Conclusion:** Overall, these data indicate that IL31RA promotes basal-like breast cancer progression and metastasis, suggesting that targeting of IL-31/IL31RA axis might be beneficial to treatment of BLBC.

## Introduction

Basal-like breast cancer (BLBC) is a cluster of breast tumor cells that characterized with low expression of estrogen receptor, progesterone receptor, and epidermal growth factor receptor 2; it frequently occurs in young women and has higher incidence rates of distant organ metastasis and recurrence than hormone receptor positive breast cancer. BLBC is insensitive to endocrine or HER2-targeted therapies with low 5-year survival rates. Few systemic treatment options exist besides chemotherapy (1–3).

Interestingly, BLBC is an inflammation-associated disease, its rapid growth and metastasis heavily relies on aberrant up-regulation of pro-oncogenic inflammatory pathways (4). For example, plenty of studies have observed that two critical cytokines, IL-6 and IL-8, are up-regulated in BLBC which are required for maintenance of breast cancer stem cell-like properties (5). Usually, BLBC has high level of infiltration of tumor associated macrophage and lymphocytes compared with other subtypes, which is correlated with the high risk for progression and distant metastasis. It is well-established that Th2-type and CD4-positive T cells are basic constitutes of the tumor microenvironment (6). Interestingly, activated Th2-type or CD4-positive T cells are able to produce Interleukin-31 (IL-31), a member of IL-6 cytokine family. IL-31 frequently activates JAK2-STAT3 signaling, promoting inflammation and allergic diseases, such as asthma and dermatitis (7). Normally, IL-31 recognizes Interleukin-31 receptor α (IL31RA), a common receptor subunit for IL-6-type cytokines on the cell membrane. IL31RA, frequently interacting with oncostatin M receptor, mediates biological or pathological functions of IL-31 (8), as evidenced by the fact that *Il31ra*-deficient mice did not develop alopecia or pruritus in response to IL-31 treatment (9). Although it is realized that expression of IL31RA mRNA is induced after stimulation with IFN-γ (10), the transcriptional regulation manner of this gene is barely known. More importantly, the pathological role of IL-31/IL31RA axis in tumor progression is largely unclear. In this study, we tried to discern the functional role of IL31RA in BLBC progression and make clear its transcription regulation manner.

Twist is a powerful epithelial-mesenchymal transition (EMT)-inducing transcription factor that activates the expression of mesenchymal-type genes and represses the epithelial phenotype (11). Twist mediated EMT process is critical for cancer cell invasion and metastasis (12, 13), and the acquisition of cancer stem-like property (14, 15). Our previous data indicated that activated Twist/BRD4 complex occupies the super-enhancer regions of oncogenes and maintains their expression. Pharmacologic inhibition of the Twist-BRD4 association by BET-specific BD inhibitors suppressed invasion, cancer stem cell (CSC)-like properties, and tumorigenicity of BLBC cells (16). However, whether Twist/BRD4 transcription complex modulates the cytokine signaling and inflammation process remains obscure.

In this study, we found that IL31RA is required for cancer stem cell (CSC)-like properties, invasion and metastasis of BLBC cells, implicating that specific targeting of this protein represents a potential therapy for BLBC treatment.

## Materials and Methods

### Reagents

Antibodies for p-STAT3 and BRD4 were purchased from Cell Signaling (Danvers, MA). Antibodies for Twist were from Abcam (Cambridge, MA). IL31RA antibody was from GeneTex (Irvine, CA). ShRNA against IL31RA and antibody against Actin were purchased from Sigma-Aldrich (St. Louis, MO). Purified human recombinant IL-31 protein was obtained from R&D systems (Minneapolis, USA). IL31RA lentivirus plasmid was purchased from GeneCopoeia (Guangzhou, China).

SiRNAs for Twist and BRD4 were obtained from Dharmacon (USA).

### Cell Culture

Breast cancer cell lines BT549 and T47D were maintained in RPMI-1640 medium with 10% fetal bovine serum. Breast cancer cell lines MDA-MB-231, MDA-MB-157, MDA-MB-453, MCF7 and BT474 were cultured using Dulbecco's Modified Eagle Medium containing 10% FBS. Penicillin/streptomycin were added to cell culture medium. All cell lines were obtained from ATCC. Stable cell clones were constructed by selection using puromycin (1 μg/mL). For siRNA transfection, cells were seeded about 1 × 10^5^ cells per well in medium with 10% FBS and transfected with siRNA using siRNA-mate reagent (GenePharma) after reaching 70% confluency.

### Western Blot

Cells were harvested and lysed, protein concentration was determined using BCA protein assay kit. Equal amounts of purified proteins were separated by SDS-PAGE electrophoresis and transferred onto PVDF membranes. Protein signals were detected using Enhanced chemiluminescence kit (FDbio science). Primary antibodies concentrations were used as following: IL31RA (1:1,000, GeneTex), p-STAT3 (1:1,000, Cell signaling), β-Actin (1:1,000, Transkgen). Anti–rabbit or anti-mouse secondary antibody (Earthox) was used at 1:5,000 dilution.

### RT-PCR Analysis

Total RNA was extracted from cells by RNeasy Mini Kit (#74104, Qiagen, Valencia, CA), and 1 μg of RNA was reverse transcribed by SuperScript^R^ III Reverse Transcriptase (#18080044) from Thermo Fisher Scientific (Waltham, MA). Real-time PCR was analyzed using Power SYBR Green Master Mix (Applied Biosystems, USA). Expression values relative to control were calculated using the ΔΔCT method. GAPDH was used as a housekeeping gene for normalization. Results were represented as relative fold change. The primers used for *IL31RA* gene were: 5′-ctggagtgactggagccaag-3′ and 5′- ctaggactggggctcctctt-3′.

### Tumorsphere

1 × 10^4^ cells were plated as single-cell suspensions on ultra-low attachment plates (Corning) in DMEM/F12 medium supplemented with 20 ng/ml EGF, 10 μg/ml insulin, 0.5 μg/ml hydrocortisone and B27. Tumorspheres were counted and taken photos after 5–7 days.

### Wound-Healing and Transwell Assay

For wound healing assay, cells were seeded at 80% confluency and cultured overnight. The culture was scratched with a 200 μl pipette tip and the wound was allowed to heal for 48 h. The reduction of area between two wound edges was calculated and quantitated. For transwell assay, each insert (8-μm pore size, Falcon) was coated with matrigel overnight. 1 × 10^5^ control or clone cells were re-suspended in 150 μl non-serum medium and seeded in the upper Boyden chamber coated with Matrigel (BD biosciences, San Jose, CA) while the bottom chambers were filled with 600 μl non-serum medium plus 100 nM LPA. After 24–48 h, un-invasive cells on the membrane apical side were removed using wet cotton swabs and the invasive cells were stained with crystal violent and counted under microscope. Typical pictures of invaded cells are shown, Scale bar, 100 μM. Statistical data (mean ± SD) for numbers of invading cells are shown.

### Chromatin Immunoprecipitation

Approximately 1 × 10^6^ control and Twist-knockdown MDA-MB-231 cells as well as JQ1-treated cells were fixed with cross-link solution and collected, ChIP assays were performed using Imprint Chromatin Immunoprecipitation Kit (Sigma, #CHP1) according to the manufacturer's instructions. Twist or BRD4 antibody-immunoprecipitated DNA was analyzed by real-time PCR. The specific primers for the *IL31RA* promoter were: 5′-GAGACAGGAAGGCAGAGTGT-3′ and 5′-TTGCGGACATTCACAGACAC-3′.

### Luciferase Reporter Assay

The human *IL31RA* gene promoter region (988 bp) was cloned and its promoter-luciferase construct was generated. HEK293T cells were seeded in 60 mm dishes and transfected with mentioned plasmids by FuGene 6 transfection reagent (Roche) for 24 h, cell lysates were extracted with passive lysis buffer (Promega, Madison, WI) and luciferase activity was measured using the Dual-Luciferase Reporter Assay System (Promega, Madison, WI). Relative luciferase activities were calculated as folds of induction compared with vector control.

### Mice Model

Balb/c female nude mice (4–6 weeks) were purchased from Animal Center of Guangdong Province. All animal experiments were approved by the Animal Care and Use Committee of Southern Medical University. Mice were housed in autoclaved, ventilated cages and provided with autoclaved water. 1 × 10^6^ MDA-MB-231 vector control cells and *IL31RA*-knockdown cells were, respectively, injected into mammary fat pad of mice (*n* = 7). Tumor growth was monitored with caliper measurements every 5 days, tumor volume was calculated according to the formula: *length* × *width*^2^*/2*. After 30 days, mice were euthanized, tumors were weighed and taken photos. For lung metastasis model, 2 × 10^5^ MDA-MB-231 cells were re-suspended in 0.1 ml PBS and injected into the tail vein. After 6 weeks, the mice were sacrificed, tumor nodules on the surface of lung were counted and quantified.

### Gene Expression Correlation Analysis

Gene expression data for breast carcinoma patients from 3 studies including GSE48390, GSE76275, and GSE93601 were downloaded from the GEO. The Pearson's and Spearman's correlation coefficients were used to quantify the correlation in expression between *Twist* and *IL31RA*. *P*-values were calculated based on testing the hypothesis of correlation coefficient equal to zero, i.e., the expressions of genes are independent.

### Statistical Analyses

Data are presented as mean ± SD. Student's *t* test (two-tailed) was used to compare two-group data which satisfy normal distribution with homogeneous variance. Multiple comparisons were analyzed by one-way ANOVA and Welch's test was used for data with unequal variance. *P* < 0.05 was considered significant. ^*^p < 0.05, ^**^p < 0.01, and ^***^p < 0.001.

## Results

### Silencing of *IL31RA* Suppresses BLBC Progression

**Figure 1:**
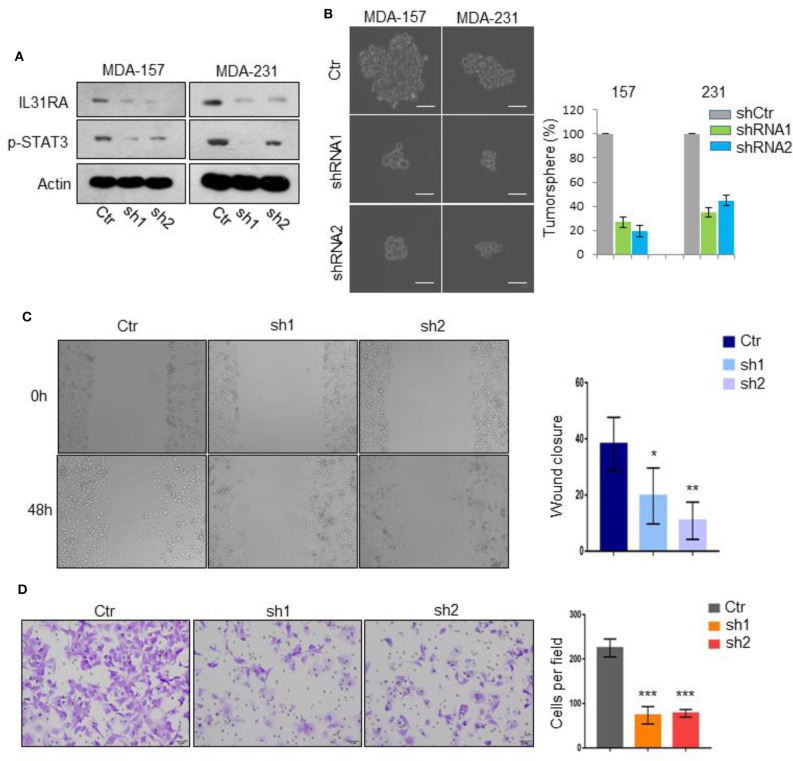
Silencing of *IL31RA* suppresses BLBC progression. **(A)** *IL31RA* gene was knocked down in MDA-MB-231 and MDA-MB-157 cells by shRNA and stable clones were constructed. Expression of IL31RA and STAT3 phosphorylation was detected by western blot. **(B)** Cancer stem cell-like properties were determined by tumorsphere assay in vector control and *IL31RA*-knockdown MDA-MB-231 and MDA-MB-157 cells. Scale bar, 100 μM. Statistical analysis (mean ± SD) for numbers of tumorsphere is shown. **(C)** Cell migration was detected by wound healing assay in vector control and *IL31RA*-knockdown MDA-MB-231 cells for 48 h. Statistical data (mean ± SD) for wound closure are shown. *indicates *P* < 0.05, **indicates *P* < 0.01. **(D)** Cell invasion was observed by transwell assay in vector control and *IL31RA*-knockdown MDA-MB-231 cells. Scale bar, 100 μM. Statistical data (mean ± SD) for invaded cells are shown. ***indicates *P* < 0.001. Scale bar, 100 μM.

To reveal the pathological role of IL31RA in BLBC, we knocked down *IL31RA* gene in MDA-MB-231 and MDA-MB-157 BLBC cell lines and constructed their stable clones. Silencing of *IL31RA* markedly repressed STAT3 tyrosine 705 phosphorylation, indicating the blockade of IL-31/IL31RA signaling (Figure 1A). Intriguingly, both *IL31RA*-knockdown clones showed the reduced cancer stem cell-like properties that determined by tumorsphere formation assay (Figure 1B). *IL31RA*-silencing also significantly inhibited the cell migratory capacity of MDA-MB-231 cells, in which about 10–20% wound closure was observed in *IL31RA*-silencing cells compared with almost 40% closure in vector control cells (Figure 1C). Consistently, remarkably weaker invasive ability was revealed in *IL31RA*-knockdown MDA-MB-231 clones than vector control cells that detected by the transwell invasion assay (Figure 1D). These results indicate that IL31RA is critical for cancer stem cell-like properties and motility of BLBC cells.

### Silencing of *IL31RA* Suppresses Tumor Growth and Metastasis *in vivo*

**Figure 2:**
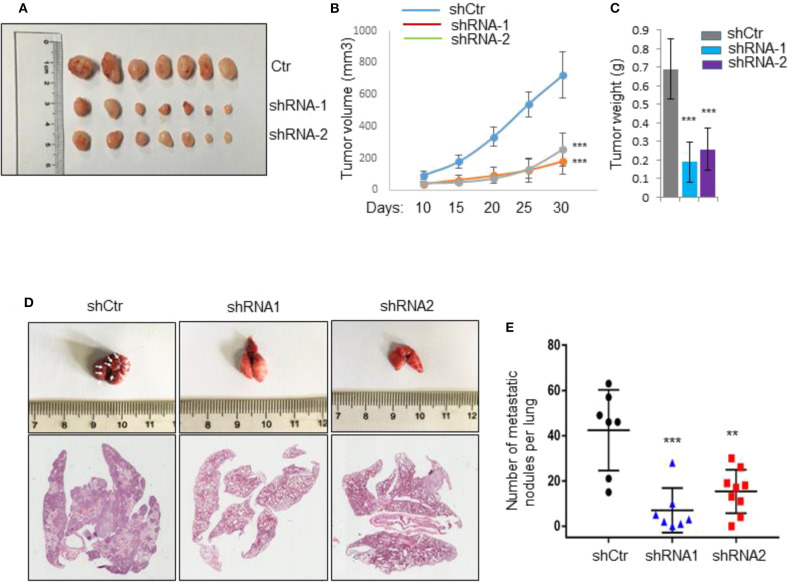
Silencing of IL31RA suppresses tumor growth and metastasis. **(A–C)** Vector control and two *IL31RA*-knockdown MDA-MB-231 clones were injected into mammary fat pad of BALB/c nude mice (*n* = 7), respectively. Xenograft tumor growth was determined by measuring tumor volume and weight. Statistical data are shown (*** indicates *P* < 0.001). **(D,E)** MDA-MB-231 vector control and *IL31RA*-knockdown cells were, respectively, injected into tail vein of BALB/c nude mice. The images of mice lung with metastatic nodules (*Left panel*) and statistical data (*Right panel*) are shown (**indicates *P* < 0.01, ***indicates *P* < 0.001). White arrows indicate the metastatic nodules.

To further study the functional roles of IL31RA *in vivo*, vector control and *IL31RA*-knockdown MDA-MB-231 clones were injected into mammary fat pad of Balb/c nude mice, respectively. After 30 days, vector control MDA-MB-231 developed into xenograft tumors with volume about 800 mm^3^, *IL31RA*-knockdown clones had significantly reduced tumor size compared with vector control cells (Figures 2A–C). Next, we performed tail vein injection to construct *in vivo* lung metastasis model in order to measure the metastatic capacity of vector control and *IL31RA*-silencing MDA-MB-231 cells. As shown, robust reduction of pulmonary nodules from *IL31RA*-knockdown clones was observed compared with vector control group (Figures 2D,E). All these data indicate that IL31RA is required for breast cancer cell invasion and metastasis.

### Overexpression of IL31RA Promotes Cancer Stem Cell-Like Properties and Cell Motility

**Figure 3:**
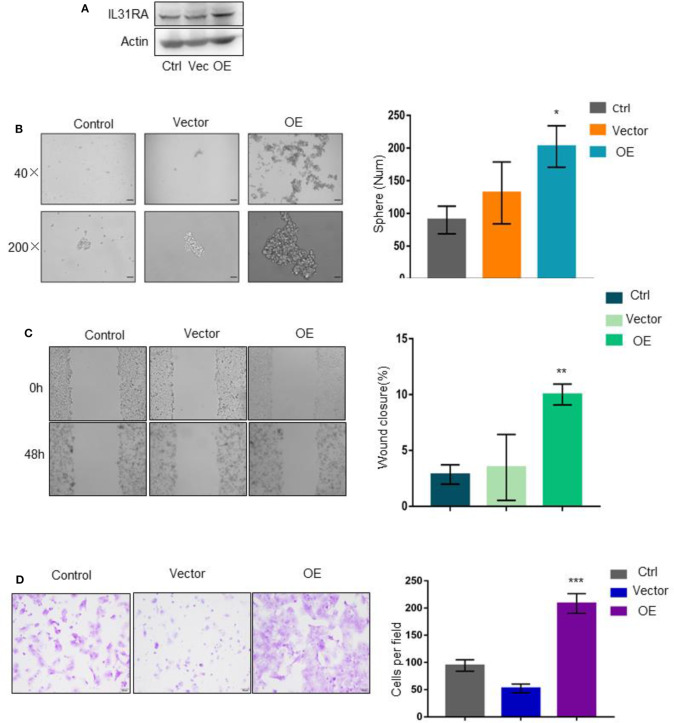
Overexpression of IL31RA promotes cancer stem cell-like properties and cell motility. **(A)** Construction of stable IL31RA over-expression clone in MCF-7 cells was determined by western blot. **(B)** Cancer stem cell-like properties were determined by tumorsphere assay in parental MCF-7, vector control and IL31RA-overexpression clone. Typical images of tumorsphere were taken at 40 and 200 magnitudes. Statistical data (mean ± SD) for numbers of tumorsphere are shown. *indicates *P* < 0.05 (OE vs. Vector). Scale bar, 100 μM. **(C)** Cell migration was detected by wound healing assay in parental MCF-7, vector control and IL31RA-overexpression clone for 48 h. Statistical data (mean ± SD) for wound closure are shown. **indicates *P* < 0.01 (OE vs. Vector). **(D)** Cell invasion was observed by transwell assay in parental MCF-7, vector control and IL31RA-overexpression clone. Scale bar, 100 μM. Statistical data (mean ± SD) for invaded cells are shown. ***indicates *P* < 0.001 (OE vs. Vector). Scale bar, 100 μM.

To verify the biological roles of IL31RA in breast cancer, we constructed IL31RA stable over-expression clone in MCF-7 luminal breast cancer cells by transfection of lentivirus-based expression plasmid (Figure 3A). As expected, this gene over-expression strongly enhanced the tumorsphere formation ability compared with vector control and parental MCF-7 cells (Figure 3B). Similarly, tumor cell migration (Figure 3C) and invasive ability (Figure 3D) were also robustly stimulated upon IL31RA over-expression. These data further indicate that IL31RA is essential for cancer stem cell-like properties, cell migration, and invasion.

### Loss of IL31RA Abrogates the Oncogenic Roles of IL-31

**Figure 4:**
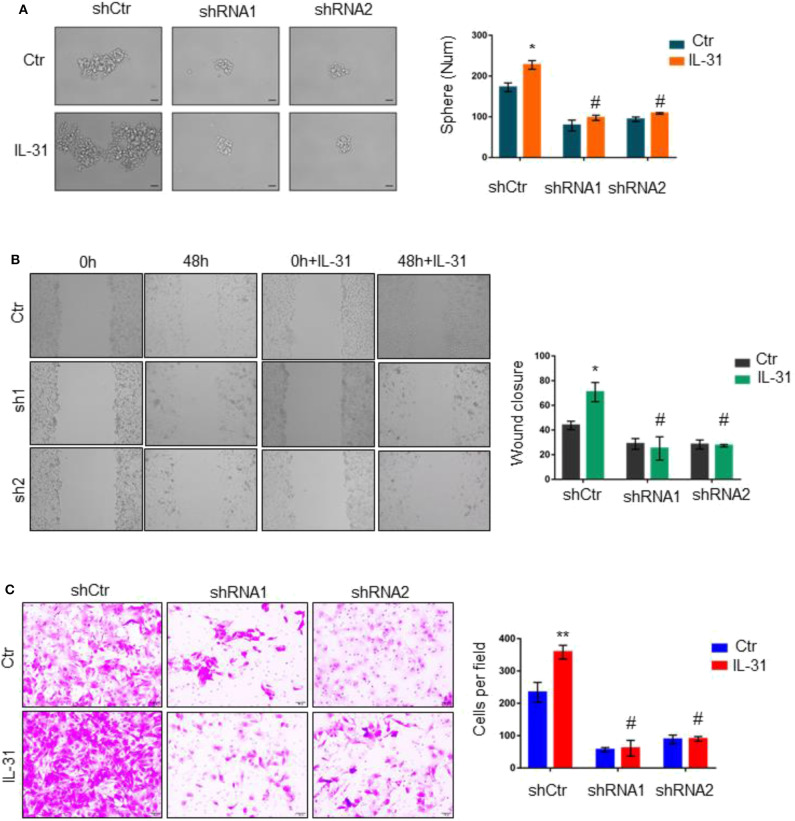
Loss of IL31RA abrogates the oncogenic roles of IL-31. **(A)** Tumorsphere assay was performed in control and IL31RA-shRNA clones of MDA-MB-231 in the absence or presence of IL-31 (10 ng/ml) treatment for 3 days. Quantitative analysis of tumorsphere numbers is shown. *indicates *P* < 0.05, ^#^indicates no significance. Scale bar, 100 μM. **(B)** Wound-healing assay was done in the absence or presence of IL-31 (10 ng/ml) treatment for 48 h. Statistical data (mean ± SD) for wound closure are shown. *indicates *P* < 0.05, # indicates no significance. **(C)** Transwell assay was conducted in *IL31RA*-knockdown and control MDA-MB-231 cells with or without IL-31 (50 ng/ml) treatment. Scale bar, 100 μM. Statistical data (mean ± SD) for invaded cells are shown. **indicates *P* < 0.01, ^#^indicates no significance.

We proceeded to investigate the functional dependence of IL-31 on IL31RA in BLBC progression. Tumorsphere formation ability of shRNA-control and *IL31RA*-knockdown clones of MDA-MB-231 was measured in the absence or presence of IL-31 (10 ng/ml) treatment. IL-31 treatment significantly enhanced the tumorsphere formation in shRNA-control MDA-MB-231 cells, but not in *IL31RA*-knockdown clones (Figure 4A). We also detected the migratory and invasive capacity of these clones with or without IL-31 treatment; similar results were observed that IL-31 induced the migration (Figure 4B) and invasion (Figure 4C) of control MDA-MB-231 cells, however, it completely lost the ability in *IL31RA*-silencing cells, indicating that IL-31 is able to promote BLBC tumor progression and its pro-oncogenic functions are strongly dependent on IL31RA.

### Twist/BRD4 Complex Induces IL31RA Expression

**Figure 5:**
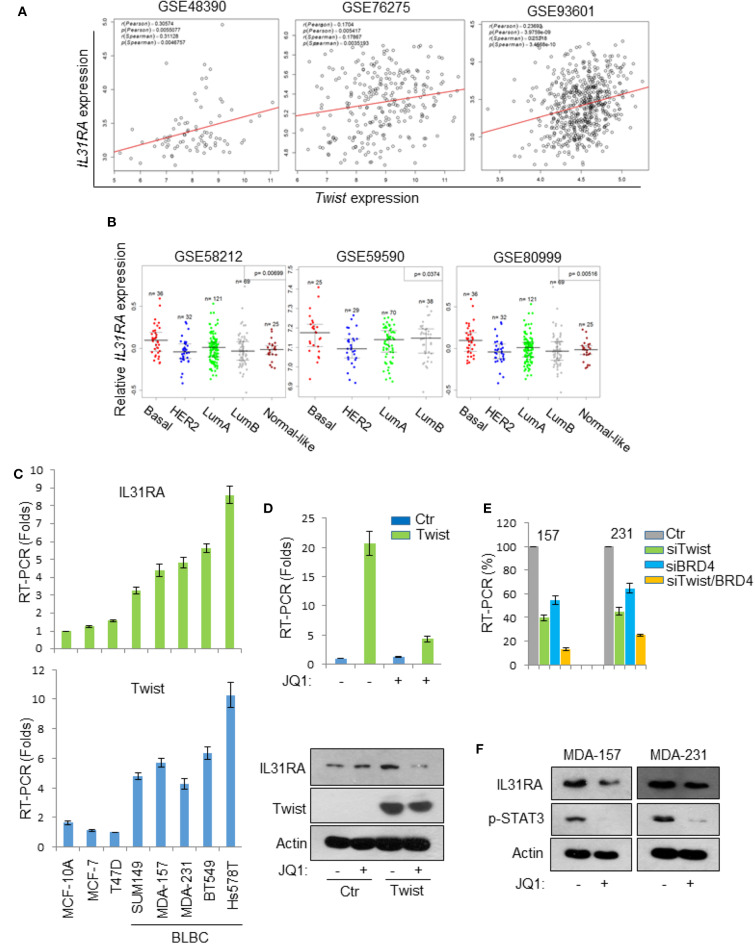
Twist/BRD4 induces IL31RA expression. **(A)** The gene expression correlation of *IL31RA* and *Twist* was analyzed based on three GEO datasets. Correlation coefficients and corresponding *p*-values are shown. **(B)** Bio-informatic analyses were performed to measure *IL31RA* mRNA level in breast cancer subtypes. **(C)** Real-time PCR experiments were conducted in a series of breast cell lines to measure the mRNA levels of *Twist* and *IL31RA*. Data presented are representative of three experiments performed as the mean ± SD. **(D)** Vector-T47D and Twist-T47D cells were treated with or without JQ1 (1 μM), real-time PCR and western blot analyses were performed to determine the expression of IL31RA. **(E)** *Twist* and/or *BRD4* were knocked down by siRNA in MDA-MB-231 and MDA-MB-157 cells; the expression of *IL31RA* mRNA was measured by real-time PCR. Data presented are representative of three experiments performed as the mean ± SD. **(F)** BLBC cells were treated with JQ1 (1 μM), the protein levels of IL31RA and p-STAT3 were detected by western blot.

Next, we sought to investigate the expression status of IL31RA in breast cancer. Three datasets from Gene expression omnibus (GEO) that contain gene expression information of breast cancer patients were analyzed; the data indicated that the mRNA level of *IL31RA* is positively correlated to that of *Twist* in breast cancer (Figure 5A). Interestingly, IL31RA was observed to have highest expression level in BLBC compared with other subtypes of breast cancer (Figure 5B). Subsequently, real-time PCR results in a cell line array also revealed that both *Twist* and *IL31RA* are highly expressed in BLBC cells (Figure 5C). Previously we generated stable Twist-overexpression clone in luminal breast cancer cell line T47D, and performed cDNA microarray analysis of vector-T47D and Twist-T47D cells (GSE53222) (16). This analysis revealed that the mRNA expression of IL31RA was greatly induced by Twist, while down-regulated by treatment of BET inhibitor JQ1. Real-time PCR assay showed that Twist over-expression robustly stimulated the mRNA level of IL31RA; pre-treatment of JQ1 markedly ameliorated this induction. Western blot results further verified this phenomenon (Figure 5D). Furthermore, knockdown of *Twist* and/or *BRD4* in two BLBC cell lines (MDA-MB-231 and MDA-MB-157) significantly decreased the expression of *IL31RA* (Figure 5E). Similarly, JQ1 treatment strongly down-regulated the protein levels of IL31RA in BLBC cells; meanwhile, JQ1 also inhibited the tyrosine 705 phosphorylation of STAT3 (Figure 5F). These results indicate that Twist/BRD4 complex induces the expression of IL31RA.

### *IL31RA* Is a Target Gene of Twist/BRD4 Complex

**Figure 6:**
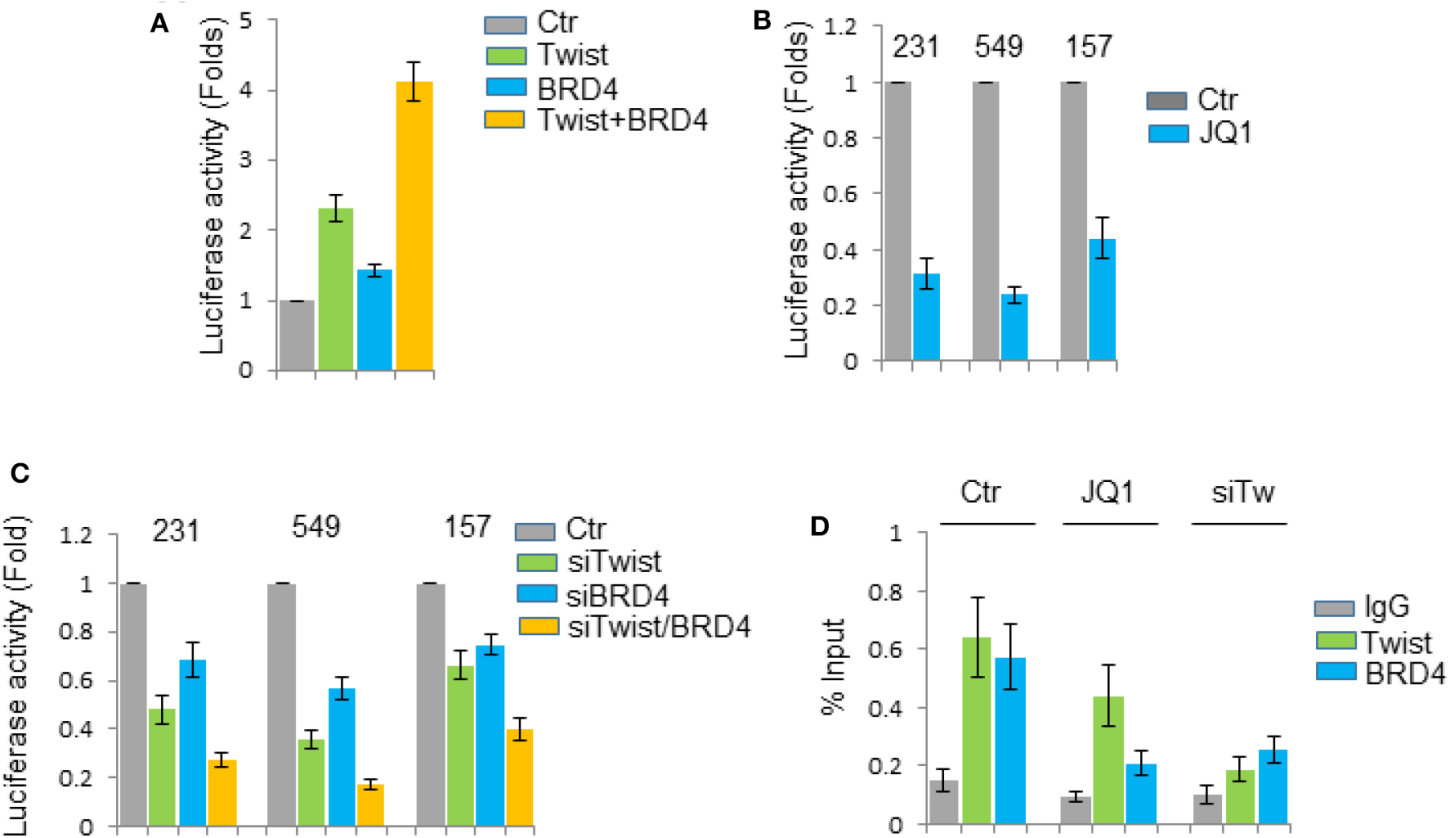
*IL31RA* is a target gene of Twist/BRD4 complex. **(A)** The *IL31RA* gene promoter luciferase activity was measured when this construct was co-expressed with Twist and BRD4 plasmids in HEK293T cells for 24 h. Statistical data (mean ± SD) for fold change are shown. **(B)** The promoter luciferase activity was measured when BLBC cells were treated with JQ1 (1 μM) for 6 h. Statistical data (mean ± SD) for fold change are shown. **(C)** The promoter luciferase activity was measured when *Twist* and/or *BRD4* was knocked down in BLBC cells. Statistical data (mean ± SD) for fold change are shown. Data presented are representative of three experiments. **(D)** Chromatin immunoprecipitation assay was performed by anti-Twist and anti-BRD4 antibodies when MDA-MB-231 cells were treated with or without JQ1 (1 μM) for 6 h, or when *Twist* was knocked down. Results were analyzed by real-time PCR. Statistical data (mean ± SD) for fold change are shown.

To determine whether *IL31RA* is a direct transcriptional target gene of the Twist/BRD4 complex, we cloned the human *IL31RA* gene promoter region and generated its promoter-luciferase construct. When this construct was co-expressed with Twist and BRD4 expression plasmids in HEK293T cells, we found that ectopic expression of Twist and/or BRD4 enhanced the *IL31RA* promoter luciferase activity (Figure 6A). Knockdown of *Twist* and/or *BRD4* greatly repressed the luciferase activity in BLBC cells (Figure 6B). BET inhibitor JQ1, which is able to disrupt the interaction between Twist and BRD4, inhibited the luciferase activity in BLBC cells as well (Figure 6C). Moreover, chromatin immunoprecipitation assay showed that Twist and BRD4 proteins associated with the *IL31RA* gene promoter; JQ1 disrupted not only the binding of BRD4 also the interaction of Twist on *IL31RA* promoter; Consistently, silencing of *Twist* weakened the binding of BRD4 to the *IL31RA* gene promoter (Figure 6D). All above data indicate that Twist/BRD4 directly regulate *IL31RA* gene transcription.

## Discussion

Plenty of studies have demonstrated the pathogenic role for IL-31 in atopic dermatitis and allergic asthma (17). In recent years, a few literatures implicated the involvement of the IL-31/IL31RA axis in cancer. The expression of IL-31 and IL31RA was found to be higher in lymph nodes from follicular lymphoma patients with grade IIIa compared with grade I/II. The elevation of IL-31/IL31RA signaling was indicated to be responsible for primary follicular lymphoma cell proliferation (18). Patients with endometrial carcinoma displayed high serum levels of IL-31, which may represent promising disease biomarkers (19). Intriguingly, the pathological roles of IL-31/IL31RA signaling in tumor progression remain largely unknown. Owing to the high rate of early-stage metastasis and lack of effective targeted therapies, it is urgent to reveal the underlying signaling molecules that determine the progression and metastasis of basal-like breast cancer cells. Our results show that the expression level of *IL31RA* is up-regulated in BLBC in comparison with other breast cancer subtypes. Silencing of *IL31RA* suppresses the cancer stem cell-like properties, migration, and invasion of BLBC cells *in vitro* as well as tumor growth and metastasis *in vivo*. These data suggest that any chemicals or antibodies that target IL-31/IL31RA axis may confer therapeutic benefit to treatment of BLBC. Recently, CIMM331 humanized anti-human IL31RA antibody was tested in a phase I/Ib study for patients of atopic dermatitis (20). We will try this antibody or screen other small inhibitors for blockade of IL-31 signaling and treatment of BLBC.

These findings also identify the transcription regulation manner of *IL31RA* gene. Our previous study indicated that the Twist/BRD4 complex represents a druggable target for treating BLBC (16). BET-specific BD inhibitors disrupt the Twist-BRD4 interaction, leading to inhibition of tumorigenicity of BLBC cells *in vitro* and *in vivo*. However, BET inhibitors are able to block the interaction of BRD4 with several other acetylated transcriptional factors or inhibit the biological functions of other BET family members, such as BRD2 and BRD3. Therefore, it is rational to develop the ways for inhibition of specific downstream signaling of Twist/BRD4 complex. Studies of Drosophila mesoderm development indicated that Twist is a master transcriptional factor which governs multiple gene transcriptional activation (21). Our microarray analysis has implicated that *IL31RA* is transcriptionally active in Twist-overexpressing cells, suggesting it is likely a target gene of Twist (16). In this study, by promoter luciferase and chromatin immunoprecipitation evidences, we identify that IL31RA transcription is directly modulated by Twist/BRD4 complex. Collectively, these data implicate that BLBC cells might employ Twist/BRD4 transcription complex to induce IL31RA expression, facilitating the utilization of IL-31 pro-oncogenic signaling and promoting the tumorigenicity of BLBC. Our study may explain how tumor cells utilize the pro-oncogenic signals derived from inflammatory microenvironment and further clarify the working mechanism of Twist/BRD4 complex.

## Data Availability Statement

The analyzed data sets generated during the present study are available from the corresponding author on reasonable request.

## Ethics Statement

The animal study was reviewed and approved by Animal Care and Use Committee of Southern Medical University.

## Author Contributions

JS conceived and designed the project, and wrote the manuscript. YH, XZ, WP, and FT carried out the experiments. LL gave advice on the manuscript.

## Conflict of Interest

The authors declare that the research was conducted in the absence of any commercial or financial relationships that could be construed as a potential conflict of interest.
